# Impact of Sleeping Habits on the Weight Loss Effect of Oral Glucagon-Like Peptide-1 Receptor Agonists Among Obese Patients: An Observational Study in Japan

**DOI:** 10.7759/cureus.87823

**Published:** 2025-07-13

**Authors:** Kazuki Takakura, Nagisa Koda, Yuji Kinoshita, Maki Oh, Muneyuki Koyama, Machi Suka

**Affiliations:** 1 Public Health and Environmental Medicine, The Jikei University School of Medicine, Tokyo, JPN; 2 Internal Medicine, UnMed Clinic Motomachi, Kanagawa, JPN; 3 Internal Medicine, The Jikei University School of Medicine, Tokyo, JPN; 4 Surgery, The Jikei University School of Medicine, Tokyo, JPN

**Keywords:** glucagon-like peptide-1 receptor agonist, obesity, overweight, semaglutide, sleeping habits

## Abstract

Although glucagon-like peptide-1 receptor agonists (GLP-1 RAs) have been used as anti-obesity agents, there are individual differences between these agents, thus making it difficult to achieve optimal treatment efficacy. This study aimed to determine the impact of sleeping habits on the weight loss effect of semaglutide, an oral GLP-1 RA, among Japanese individuals with obesity. Data were collected from March 2022 to October 2024. A total of 367 Japanese adults (30.5% men and 69.5% women) were included for analysis. Among them, 83.9% had a body mass index (BMI) between 25 and 35 kg/m^2^, and 16.1% had a BMI of 35 kg/m^2^ or more. The percentage change from baseline in body weight was assessed every three months and at the end of the follow-up period. The assessments were conducted in accordance with the Japanese guidelines for the management of obesity. Logistic regression was used to assess the associations between the therapeutic efficacy of semaglutide and potentially related factors, including sleeping habits. Participants who exhibited improvements in sleeping time or sleep quality during the treatment period were considered to have experienced sufficient treatment effects. These findings suggest that getting enough sleep may improve the weight loss effect of GLP-1 RAs in real-world settings.

## Introduction

The number of individuals with overweight and obesity has been considerably increasing worldwide. Therefore, more rigorous management is needed in many countries, including Japan. Obesity is a major risk factor for the deterioration of lifestyle diseases, cardiac infarction, stroke, and cancers in various organs; therefore, the World Health Organization is now playing a central role in accelerating the prevention of obesity [1-3]. Moreover, glucagon-like peptide-1 receptor agonists (GLP-1 RAs) have been clinically approved as antidiabetic agents and have been shown to suppress appetite, which has resulted in their use in clinical settings as anti-obesity drugs [4]. However, not everyone who is treated with GLP-1 RAs experiences weight loss, because there are individual differences in the effects of GLP-1 RAs [5-8]. Indeed, variability in treatment outcomes is an important topic for healthcare professionals who have obese patients struggling with weight loss. Hence, detailed information regarding how to obtain sufficient weight loss efficacy of GLP-1 RA is indispensable to improve the success rate in more cases.

Recently, several investigations have revealed that a lack of sleep is a significant risk factor for obesity, and the underlying mechanisms have also become clear [9-12]. In particular, an association has been observed between appetite-related hormones, which are influenced by the sleeping state, and obesity [13-17]. Therefore, herein, we investigated the impact of sleeping habits on the weight loss effect of semaglutide, a daily oral GLP-1 RA, among Japanese individuals with obesity. Moreover, we also evaluated whether improving sleeping habits, regardless of the use of sleeping pills, can yield better treatment outcomes with semaglutide.

Furthermore, treatment progress with semaglutide was tracked and evaluated over time to perform a longitudinal evaluation of treatment outcomes with semaglutide, considering that obese patients frequently experience body weight plateau or regain after successful initial weight loss, which makes sustained weight management much more challenging [18,19].

The occurrence of obesity is multifactorial and involves genetic and environmental factors, but it is ultimately due to a long-term imbalance in energy intake and energy expenditure [20,21]. Accordingly, to perform a comprehensive assessment of the factors that influence the weight loss efficacy of semaglutide in addition to sleeping habits, various other factors, such as sex, age, medical history, dietary habits, smoking and alcohol habits, exercise routine, and stress level, were assessed via secondary analysis.

## Materials and methods

Study design and population

A total of 870 patients who started treatment with semaglutide at UnMed Clinic Motomachi between March 2022 and October 2024 were potentially eligible for inclusion in this observational cohort study. All demographic and clinical data were retrospectively extracted from electronic medical records on the basis of self-reported information. In a preliminary questionnaire, participants were asked about their medical history, dietary habits, drinking and smoking habits, degree of stress, and regular exercise routine with reference to the latest guidelines for the management of obesity disease from the Japan Society for the Study of Obesity (JASSO) [22]. Clinical data, such as age, sex, height, weight, and body mass index (BMI), were also collected at baseline and during follow-up. Additionally, sleeping hours and sleep quality before initiating treatment and during treatment were also verified for all the participants by self-report. Sleep quality was assessed by dividing the participants into two groups: sufficient sleep quality and poor sleep quality. The degree of stress was also collected by self-report and assessed by dividing the participants into three groups: none, moderate, and high. In accordance with the same guidelines, the percentage change from baseline in body weight was assessed after three months, six months, and the final follow-up period from treatment initiation [22].

In terms of the dosage of semaglutide, the attending physician initially started with 3 mg as the minimum dosage and gradually increased to 7 mg and 14 mg as needed, considering not only its appetite-suppressing effect but also its side effects.

Participants were provided with informed consent for study participation and relevant information, including their right to withdraw at any time. The study protocol was approved by the Ethics Committee of UnMed Clinic Motomachi, Kanagawa, Japan (authorization number: UM23-01). The review board approved and waived the need for written informed consent from the participants because of the retrospective, non-interventional nature of this study. The study followed the recommended guidelines of the Declaration of Helsinki [23]. This study was reported in accordance with the Strengthening the Reporting of Observational Studies in Epidemiology (STROBE) reporting guideline [24].

**Figure 1:**
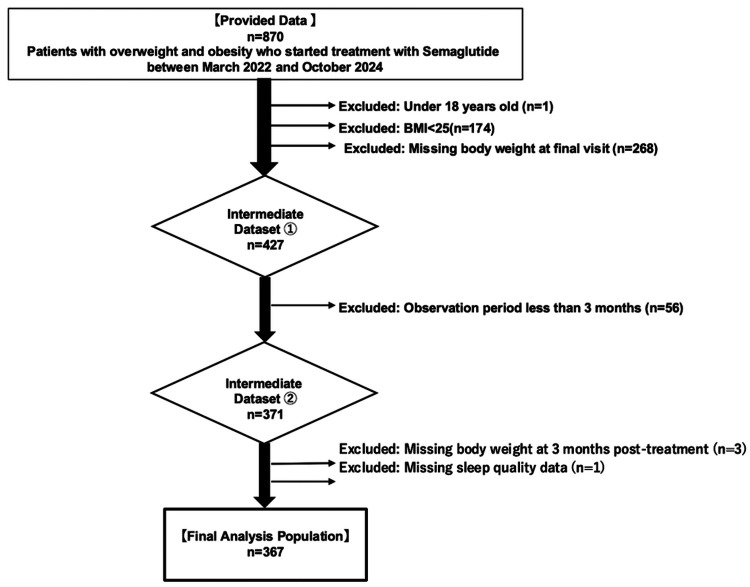
Flowchart of the study participants The reasons for screening failure and discontinuation from the study are indicated. BMI: body mass index

For the evaluation of therapeutic efficacy, 503 censored patients were excluded because of being under the age of 18, lack of necessary information, a BMI less than 25, or a tracking period of less than three months. In addition, participants who did not provide consent to participate in the study were excluded. Ultimately, a total of 367 patients with obesity who received semaglutide for more than three months were included in the analyses, as shown in Figure 1. A summary of the data collected from the study participants is presented in Table 1.

**Table 1:** Demographic and clinical characteristics of the study participants

Parameters		Number	%
Sex	Male	112	30.5%
	Female	255	69.5%
Age	18-29 years	31	8.4%
	30-59 years	298	81.2%
	60+ years	38	10.4%
Body mass index	25-34 kg/m^2^	308	83.9%
	35+ kg/m^2^	59	16.1%
Alcohol habits	Yes	91	24.8%
Smoking habits	Yes	55	15.0%
Exercise habits	Yes	143	39.0%
Breakfast	No	46	12.5%
Dinner time	After 9 PM	58	15.8%
Interval between dinner and bedtime (hours)	Less than 3	25	6.8%
Stress levels	0	140	38.1%
	1	116	31.6%
	2	111	30.2%
Medical history	Metabolic diseases	158	43.1%
	Cancers	11	3.0%
	Mental disorders	45	12.3%
	Sleep apnea	21	5.7%
Sleeping hours before treatment	Less than 6	115	31.3%
Sleep quality before treatment	Poor	160	43.6%
Sleeping pills	Lemborexant	51	13.9%
	Others	36	9.8%
Adverse events	Yes	26	7.1%

Weight loss criteria for Japanese individuals with obesity

Obesity is defined on the basis of BMI, which is calculated as the mass in kilograms divided by the square of the height in meters (kg/m^2^). According to the guidelines for the management of obesity from JASSO, obesity is defined as excessive fat storage in adipose tissue associated with a BMI of 25 kg/m^2^ or greater in Japan [22,25], and a BMI of 35 kg/m^2^ or greater is regarded as high-degree obesity. The BMI threshold of Japanese individuals is lower than that of individuals in Western countries (30 kg/m^2^ or greater), as Japanese individuals are susceptible to obesity-related diseases at lower BMIs [26-28]. Consequently, a BMI of 25 kg/m^2^ or greater is considered an appropriate threshold for Japanese individuals with obesity, although patients with a BMI between 25 and 30 kg/m^2^ are considered overweight according to Western standards.

Additionally, the initial weight loss goals are defined in the same guidelines as a decrease of 3% or more in initial weight in the first three months [22]. Thus, the evaluation of weight decrease in this study was performed according to the guidelines in a way that is appropriate for Japanese obese individuals.

Outcome measures

The percentage change from baseline in body weight was assessed once every three months and at the end of the follow-up period from treatment initiation. Additionally, all of the participants whose treatment progressed with semaglutide for up to 20 months were tracked and evaluated over time. We also investigated the associations between several clinical factors, including sleeping habits, and the weight loss efficacy of semaglutide in Japanese patients with obesity.

Sleeping habits during the treatment period

With respect to the sleeping hours reported by all of the study participants, we selected six hours, which is less than the hours recommended by the National Sleep Foundation Report, as the cutoff value in this analysis [29]. With respect to the sleeping situation, we confirmed whether the participants were taking sleeping pills or not, including before starting treatment.

Statistical analysis

All statistical analyses were performed via SAS V.9.4 software (SAS Institute, Cary, NC). The chi-square test was used to compare the distributions of categorical variables. Multiple logistic regression analysis was performed to identify factors associated with treatment efficacy with semaglutide. Odds ratios (ORs) and 95% confidence intervals (CIs) for treatment outcomes were calculated via multivariate adjustment. This multivariate analysis was applied to target changes in sleeping habits during the treatment period as an explanatory variable and the weight loss efficacy of semaglutide as a response variable. The influence of changes in sleeping habits, including sleeping hours and sleep quality during the treatment period, on the weight loss efficacy with semaglutide was analyzed via three adjusted models along the treatment time axis: Model 1, at the initial visit, reflecting before initiating treatment; Model 2, changes in sleeping habits during treatment period; and Model 3, therapeutic interventions for sleeping habits involving the use of sleeping pills under treatment with semaglutide. Sex, age, BMI, alcohol and smoking habits, exercise habits, habit of eating breakfast, interval between dinner and bedtime, and stress level were included as fixed factors. The threshold for statistical significance was set at p<0.05.

## Results

Participant characteristics

A total of 367 Japanese participants with obesity, regardless of the presence or absence of diabetes, who received semaglutide at UnMed Clinic Motomachi between March 2022 and October 2024, were included in the analyses (Figure 1). The self-reported characteristics of the study population are summarized in Table 1. There was a greater predominance of female to male subjects, i.e., 69.5% to 30.5%, respectively. The majority of the participants (81.2%) belonged to the age group ranging from 30 to 59 years. Among them, 83.9% have obesity (25 kg/m^2^ < BMI < 35 kg/m^2^), and 16.1% have high-degree obesity (BMI ≥ 35). In terms of dietary patterns, 46 (12.5%) participants usually skip breakfast, 58 (15.8%) participants have late dinner after 9 PM, and 25 (6.8%) sleep within three hours of having dinner. According to the results of the questionnaire, the level of stress was 0 (none) in 38.1%, 1 (medium) in 31.6%, and 2 (high) in 30.2% of the study participants. Additionally, 115 (31.3%) participants slept less than six hours, and 160 (43.6%) participants experienced poor-quality sleep at the beginning of treatment. The frequency of adverse events led to the discontinuation of semaglutide in 26 (7.1%) participants, primarily gastrointestinal problems; no serious events occurred, and a total of 87 (23.7%) were taking any sleeping pills during the treatment period.

Semaglutide had both immediate and long-acting effects on weight reduction in Japanese obese individuals

**Figure 2:**
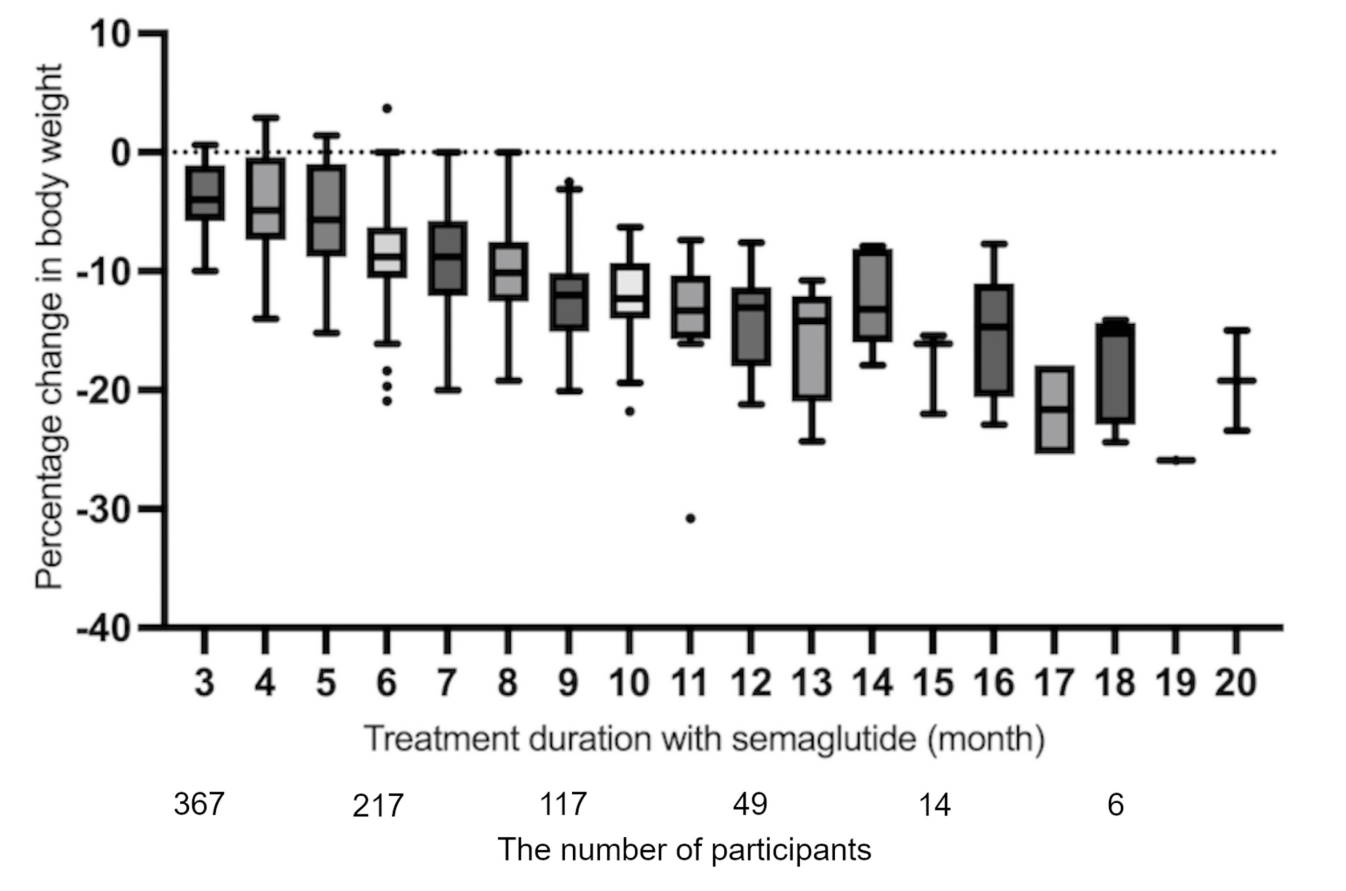
Time series graph showing the chronological evaluation of weight reduction with semaglutide

At three months, the average percentage change from baseline in body weight was 4.6%, ranging from -2.9% to 14.8%. At six months, the average percentage change from baseline in body weight was 8.6%, ranging from -3.7% to 22.2% (Table 2). As shown in Table 3, 284 (77.4%) study participants achieved a weight reduction of at least 3% at three months of treatment with semaglutide. Additionally, 187 (86.2%) participants at six months successfully achieved sustained weight loss with semaglutide. All of the achievers at six months were able to maintain weight loss throughout the entire treatment period. Clinically meaningful weight reduction at an early stage (by three months) and subsequent weight management were achieved by long-term administration of semaglutide in approximately 80% of the study participants. Conversely, nonachievers who could not lose weight at an early stage ended up failing by the end of the observation period. Overall, sustained weight reduction with semaglutide was observed over the follow-up period of up to 20 months in this study (Figure 2). In short, a good start with semaglutide is an important step in long-term, successful weight management.

**Table 2:** Average percentage change in the body weight of the study participants

Time point	Median (range), %
After 3 months of treatment	4.6 (-2.9 to 14.8)
After 6 months of treatment	8.6 (-3.7 to 22.2)

**Table 3:** Longitudinal data of the weight loss success rate with semaglutide

Time point	Total	Achiever	Rate
At 3 months of treatment	367	284	77.4%
At 6 months of treatment	217	187	86.2%
At the last observation	176	151	85.8%

Severe stress and late dinner have a negative influence on the weight loss effect of semaglutide

In the evaluation of factors related to a lack of success in weight loss after three months of treatment with semaglutide, a high level of stress significantly interfered with the success rate of weight reduction for patients with obesity after three months of treatment with semaglutide (p<0.001). Moreover, late dinner after 9 PM and less than three hours from dinner to bed also resulted in a reduced therapeutic response to semaglutide (p<0.001) (Table 4). Conversely, differences in sex, age, drinking and smoking habits, habit of eating breakfast, and medical history had no significant influence on the weight loss effect of semaglutide, suggesting that there is no difference in the outcomes between diabetic and non-diabetic participants. Interestingly, the degree of obesity was also not correlated with the weight loss success rate after three months of treatment with semaglutide, whereas exercise habits on a daily basis were significantly related in the study population, as expected (p=0.017) (Table 4).

**Table 4:** Factors related to the weight loss success rate after three months of treatment

Parameters			Total	Achiever	Rate	Chi-square	p
Sex	Male		112	80	71.4%	3.267	0.071
	Female		255	204	80.0%		
Age	18-29 years		31	28	90.3%	3.425	0.180
	30-59 years		298	226	75.8%		
	60+ years		38	30	78.9%		
Body mass index	25-34 kg/m^2^		308	237	76.9%	0.208	0.648
	35+ kg/m^2^		59	47	79.7%		
Alcohol habits	No		276	214	77.5%	0.015	0.904
	Yes		91	70	76.9%		
Smoking habits	No		312	240	76.9%	0.253	0.615
	Yes		55	44	80.0%		
Exercise habits	No		224	164	73.2%	5.712	0.017
	Yes		143	120	83.9%		
Breakfast	No		321	253	78.8%	3.001	0.083
	Yes		46	31	67.4%		
Dinner time	Before 9 PM		309	255	82.5%	29.517	<0.001
	After 9 PM		58	29	50.0%		
Interval between dinner and bedtime (hours)	More than 3		342	274	80.1%	21.424	<0.001
	Less than 3		25	10	40.0%		
Stress levels	0		140	125	89.3%	27.893	<0.001
	1		116	91	78.4%		
	2		111	68	61.3%		
Medical history	Metabolic diseases	No	209	162	77.5%	0.005	0.946
		Yes	158	122	77.2%		
	Cancers	No	356	275	77.2%	-	1.000
		Yes	11	9	81.8%		
	Mental disorders	No	322	253	78.6%	2.115	0.146
		Yes	45	31	68.9%		
	Sleep apnea	No	346	270	78.0%	-	0.280
		Yes	21	14	66.7%		
Sleeping hours before treatment (hours)	More than 6		252	210	83.3%	16.263	<0.001
	Less than 6		115	74	64.3%		
Sleep quality before treatment	Good		207	177	85.5%	17.902	<0.001
	Poor		160	107	66.9%		
Adverse events	No		341	265	77.7%	0.297	0.586
	Yes		26	19	73.1%		

The weight loss effect of semaglutide is greater in female patients than in male patients, but there are no age differences

Similarly, female patients with obesity are more likely to experience weight loss with semaglutide, as shown in Table 5 (OR: 0.52, 95% CI: 0.28-0.96). In contrast, age was not a factor related to weight loss failure after three months of treatment with semaglutide (OR: 1.00, 95% CI: 0.98-1.03). These data are promising for obese women who are considering weight loss treatment and suggest that it is never too late to start weight loss treatment, even for patients aged 60 and above.

**Table 5:** Factors related to lack of success in losing weight after three months of treatment OR: odds ratio, CI: confidence interval, Model 1: at the initial visit, Model 2: changes in sleeping habits during the treatment period, Model 3: the use of sleeping pills

Parameters		Model 1		Model 2		Model 3	
		OR	95% CI	OR	95% CI	OR	95% CI
Sex	Female	0.521	0.282-0.961	0.505	0.261-0.978	0.511	0.274-0.955
Age		1.001	0.976-1.027	1.012	0.985-1.040	1.006	0.980-1.033
Body mass index		0.987	0.928-1.049	0.981	0.917-1.049	0.978	0.917-1.044
Alcohol habits	Yes	0.942	0.488-1.818	0.965	0.469-1.985	1.012	0.520-1.969
Smoking habits	Yes	0.846	0.379-1.889	0.626	0.255-1.540	0.779	0.345-1.759
Exercise habits	Yes	0.697	0.383-1.269	0.974	0.509-1.864	0.767	0.417-1.412
Breakfast	Yes	0.727	0.341-1.551	1.476	0.614-3.550	0.807	0.372-1.749
Interval between dinner and bedtime (hours)	Less than 3	4.132	1.586-10.761	3.748	1.394-10.076	4.208	1.610-11.001
Stress levels	0	1.000	Reference	1.000	Reference	1.000	Reference
	1	2.007	0.968-4.164	1.917	0.888-4.141	2.043	0.972-4.293
	2	3.759	1.822-7.753	2.746	1.248-6.045	3.561	1.701-7.454
Sleeping hours before treatment	More than 6	0.486	0.275-0.859	-	-	0.431	0.238-0.777
Changes in sleeping hours	Less than 6	-	-	1.000	Reference	-	-
	To improve under treatment	-	-	0.147	0.056-0.383	-	-
	More than 6	-	-	0.231	0.112-0.479	-	-
Sleep quality before treatment	Good	0.589	0.328-1.059	-	-	0.454	0.243-0.846
Changes in sleep quality	Poor	-	-	1.000	Reference	-	-
	To improve under treatment	-	-	0.210	0.071-0.621	-	-
	Good	-	-	0.224	0.100-0.501	-	-
Sleeping pills	Lemborexant	-	-	-	-	0.448	0.186-1.081
	Others	-	-	-	-	2.878	1.251-6.618
Adverse events	Yes	1.237	0.449-3.609	0.963	0.322-2.876	1.245	0.441-3.512

Sleep extension enhances the weight loss effect of semaglutide in obese patients

Finally, we investigated the impact of getting enough sleep with or without the use of sleeping pills under treatment with semaglutide. The evaluation was conducted with consideration of the changes in sleeping habits during the treatment period, as shown in Models 1-3 in Table 5. In the comparison of sleeping habits, patients who originally had more than six hours of sleep (OR: 0.49, 95% CI: 0.28-0.86) and had sufficient sleep quality by using sleeping pills (OR: 0.45, 95% CI: 0.24-0.85) had better weight loss outcomes with semaglutide for three months than those who did not (Table 4). Additionally, patients who could extend their sleeping time to more than six hours (OR: 0.15, 95% CI: 0.06-0.38) or improve their sleep quality during the treatment period (OR: 0.21, 95% CI: 0.07-0.62) successfully maintained the therapeutically effective level of semaglutide for three months, regardless of whether lemborexant, a newer orexin receptor antagonist that promotes sleep, was used (Table 5). These results show the positive impact of sleeping habits on weight loss treatment.

## Discussion

This real-world observational study elucidated the impact of sleeping habits on the weight loss efficacy of semaglutide in Japanese patients with obesity. It is recognized that having more than six hours of sleep a day and enough good-quality sleep play essential roles in achieving the expected treatment outcomes for weight reduction. Moreover, improving sleeping habits during treatment enhances the weight loss efficacy of semaglutide, which has both immediate and long-acting effects on weight reduction. These findings suggest that the therapeutic effect of oral GLP-1 RA can be improved by providing enough sleep, leading to sustained weight loss management for obese patients. Related factors, such as severe stress, late dinner after 9 PM, and less than three hours from dinner to bedtime, significantly interfered with the efficacy of weight loss treatment on the basis of their direct influence on sleeping habits.

With respect to both the immediate and long-lasting effects of semaglutide for weight reduction, obese people frequently experience a body weight plateau by six months after starting self-managed hypocaloric diets, even if the initial weight loss is successful [18,19]. Since long-term maintenance of weight loss was originally much more difficult, semaglutide can become a reliable support for obese individuals to overcome this challenge. Because its impact extended beyond just the weight loss phase, its involvement in weight reduction continues in the subsequent maintenance phase. Consequently, semaglutide is suitable as a first-choice drug for weight management on the basis of its favorable outcomes in many cases and its adverse events occurring in only a few cases in the study population.

A previous study reported that sleep and meal timing may influence food intake and appetite-related hormones in overweight and obese adults [30]. Another study confirmed that short sleep duration or poor sleep quality was associated with weight regain after successful weight loss in obese adults [31]. Additionally, a Japanese multicenter study revealed that shorter sleep duration is a significant risk factor for obesity [9]. Given these previous reports, our results provide further evidence that obese patients should improve their sleep habits regardless of the use of sleeping pills when starting treatment with GLP-1 RAs. To our knowledge, this is the first study showing that sleep amelioration during the treatment period leads to improved weight reduction with oral GLP-1 RA. The underlying mechanisms explaining the link between lack of sleep and diet intake are a homeostatic control through changes in the appetite-related hormones, including leptin and ghrelin [32]. Their hormones with GLP-1 have a complementary influence on the control of appetite. Thus, better sleep is recognized as an advantageous condition for obese patients receiving weight loss treatment.

Interestingly, obese women were more likely to experience weight loss with semaglutide, whereas there was no significant age difference in the therapeutic response according to our data. Regarding sex differences in the therapeutic efficacy of semaglutide, our data are consistent with a previous report that obese women can lose more weight than obese men when treated with GLP-1RAs for weight loss [33]. Estrogen, a female hormone that reduces food intake by potentially increasing the sensitivity of the female brain to anorexigenic peptides such as GLP-1, may be a factor influencing sex differences [34]. With respect to the lack of an age difference in the therapeutic efficacy of semaglutide, a recent study demonstrated that total daily energy expenditure is stable in adults aged 20-60 years, although it begins to decline slowly after the age of 60 years [35]. The influence of semaglutide on the suppression of total energy intake may be shown directly in the treatment outcome, which is based on almost the same total energy expenditure. Taken together, these results can potentially provide grounds for future obese patients who wish to undergo weight loss treatment with GLP-1 RAs.

This study has several limitations. First, the limited sample size and the single study site of the study participants are potential sources of selection bias. Second, this study exclusively targeted Japanese individuals with obesity and followed the Japanese guidelines for the management of obesity, which include different BMI thresholds (over 25 kg/m^2^) compared with those used in Western countries (over 30 kg/m^2^). Therefore, patients who were overweight (25 kg/m^2^ < BMI < 30 kg/m^2^) according to Western standards were also included in this study. Therefore, there might be different interpretations of the analyses based on race or ethnicity. Third, this study relies on self-reported data for variables such as sleep quality and stress levels. Because both sleep quality and stress levels are basically subjective, these evaluations are commonly conducted by the self-report method [36,37]. Objective measures of sleep quality, such as polysomnography, are expensive, time-consuming, and not readily available to most clinicians in our daily routine. Indeed, several self-report questionnaires, such as the Pittsburgh Sleep Quality Index and Insomnia Severity Index, have been developed and reported good internal reliability and validity [36]. The evaluation of stress level by self-reports illuminates the duration and severity of the psychological response to stressors. However, it is certainly true that biomarkers capture a more objective measure of stress and create a deeper understanding of the biological response to chronic and acute stress [37]. Finally, we focused only on oral GLP-1 RA as a therapeutic intervention to reduce the complexity of the analysis, even in the current situation in which a number of weight loss medications, including tirzepatide, a dual glucose-dependent insulinotropic polypeptide /GLP-1 receptor agonist, are available. Further comprehensive investigations with validated objective measures of sleep quality and stress in larger populations are needed to obtain positive evidence of our findings.

## Conclusions

This observational study shows that sleeping habits can have a positive effect on weight loss efficacy with oral GLP-1 RA in obese patients in real-world clinical practice, particularly women, regardless of age. Moreover, improving sleeping habits during treatment enhances the weight loss efficacy of semaglutide. Conversely, severe stress and late dinner have a negative influence on the weight loss effect of semaglutide. Because of its immediate and long-term effects and relatively low risk of adverse events, semaglutide is well suited for weight management as a first-choice drug for obese individuals with uncontrolled weight gain. Multicenter studies are required in the future to support the current findings and thoroughly investigate the underlying biological mechanisms linking sleep and semaglutide efficacy.
